# Pre-Clinical Development of an Adenovirus Vector Based RSV and Shingles Vaccine Candidate

**DOI:** 10.3390/vaccines11111679

**Published:** 2023-11-02

**Authors:** Lawrence Petherbridge, Charlotte Davis, Angela Robinson, Thomas Evans, Sarah Sebastian

**Affiliations:** Vaccitech Ltd., Harwell OX11 0DF, UK; lawrence.petherbridge@vaccitech.co.uk (L.P.); angela.robinson@vaccitech.co.uk (A.R.); tom.evans@vaccitech.co.uk (T.E.)

**Keywords:** shingles, herpes zoster, chimpanzee adenovirus, glycoprotein E, prefusion F protein, viral vectored vaccine, varicella zoster virus, respiratory syncytial virus

## Abstract

Respiratory syncytial virus (RSV) infection and shingles are two viral diseases that affect older adults, and a combined vaccine to protect against both could be beneficial. RSV infection causes hospitalisations and significant morbidity in both children and adults and can be fatal in the elderly. The RSV fusion (F) envelope glycoprotein induces a strong RSV-neutralising antibody response and is the target of protective immunity in the first RSV vaccine for older adults, recently approved by the FDA. An initial childhood infection with the varicella zoster virus (VZV) results in chickenpox disease, but reactivation in older adults can cause shingles. This reactivation in sensory and autonomic neurons is characterized by a skin-blistering rash that can be accompanied by prolonged pain. The approved protein-in-adjuvant shingles vaccine induces VZV glycoprotein E (gE)-fspecific antibody and CD4^+^ T cell responses and is highly effective. Here we report the evaluation of RSV/shingles combination vaccine candidates based on non-replicating chimpanzee adenovirus (ChAd) vectors. We confirmed the cellular and humoral immunogenicity of the vaccine vectors in mice using T cell and antibody assays. We also carried out an RSV challenge study in cotton rats which demonstrated protective efficacy following a homologous prime-boost regimen with our preferred vaccine candidate.

## 1. Introduction

Respiratory syncytial virus (RSV) causes acute respiratory infections (ARI) in children under 5 [1] and is also common in older adults, with risk of serious complications increasing with age. RSV has been identified as a significant viral pathogen in older adults (≥65 years) with ARI, with one study finding that it accounted for 11% of hospitalisations for pneumonia, 11% for chronic obstructive pulmonary disease (COPD), 5% for congestive heart failure and 7% for asthma [2]. It is estimated that in older adults there are 14,000 in-hospital deaths per year from RSV infections globally [3]. Consequently, there is a substantial economic burden associated with RSV-related hospitalisations worldwide, with several studies in the United States estimating costs per patient that range from US $8241 per hospitalisation [4] to US $23,194 per patient per year [5] in patients 65 years and older [4,5,6,7].

Two prophylactic products have recently been granted FDA approval for use in older adults: Arvexy (RSV-preF protein in AS01 adjuvant) from GSK [8,9] and Abrysvo (a bivalent unadjuvanted preF protein vaccine) from Pfizer [10,11]. Additional RSV vaccine candidates are currently being assessed in clinical trials. Among these are several viral vector candidates, based on modified vaccinia Ankara (MVA) as well as chimpanzee and human adenoviruses: Bavarian Nordic are trialling MVA-BN^®^ RSV, an MVA-based vector expressing five RSV proteins (fusion protein, glycoprotein G, nucleoprotein and transcription elongation factor (M2-1) from subtype A and glycoprotein G from subtype B), which showed an efficacy of 79% in preclinical studies [12,13,14,15]. GSK have developed a chimpanzee adenoviral viral vector (ChAd155) expressing RSV fusion (F), nucleocapsid and M2-1 [16], which is being evaluated in clinical trials in both adults and children [17,18,19]. A human adenoviral vector (Ad26) expressing preF [20] was until recently in Phase 3 trials [21]; however, further development of this vector has been discontinued due to the approval by the FDA of Arexvy and Abrysvo [22]. Recently, Moderna have announced an mRNA-based RSV vaccine (mRNA-1345) which is currently in Phase 3 trials in older adults [23].

Like RSV, shingles, or herpes zoster (HZ), also affects older adults. HZ results from the reactivation of latent varicella zoster virus (VZV) in adults and causes painful neurocutaneous disease that can persist for months or years as post-herpetic neuralgia (PHN) [24]. Since most adults (~95%) have been exposed to VZV in the form of a childhood chickenpox infection and therefore have latent VZV in the sensory ganglia, they are at risk of developing HZ later in life [25]. In 2015, HZ and PHN incurred an estimated €271.21 million (approximately US $301.04 million) in the United Kingdom in direct medical costs and US $2.6 billion in the USA in 2020 [25,26,27].

VZV-specific immune responses acquired after childhood chickenpox infection have been shown to wane with age; immunosuppression resulting from disease or medication can also cause VZV immunity to fall [28,29,30,31]. Both scenarios can result in reactivation of VZV. Reactivation prior to this is thought to be prevented via VZV-specific CD4^+^ and CD8^+^ T cell memory responses that develop following childhood exposure [32]. Immunosenescence in adults over 50 years increases the risk of developing HZ, reaching 50% in those over 80 years of age who are unvaccinated [31]. However, protection can be restored via vaccination [33,34,35].

There are currently two main HZ vaccines that have been granted regulatory approval [25], zoster vaccine live (ZVL or Zostavax from Merck & Co., Inc.), which is a live attenuated vaccine [36,37] and the adjuvanted recombinant protein vaccine (RZV or Shingrix from GlaxoSmithKline, Inc), which consists of VZV envelope glycoprotein E (gE) in AS01B adjuvant [36,38,39]. A third vaccine (SKYZoster, live attenuated VZV) was approved for use in South Korea in 2017 and more recently in Malaysia [40].

A review of the effectiveness of current vaccines in preventing HZ estimated that Zostavax prevented HZ in 45.9% (95% confidence interval (CI) 42.2–49.4; seven studies) of adults and that the recombinant vaccine was protective in 79.2% (95% CI 57.6–89.7) of adults [41]. A comparison of the efficacy of Zostavax and Shingrix in preventing HZ in subjects aged ≥60 and ≥70 years showed that Shingrix had a higher efficacy in both groups and that Zostavax efficacy decreased in those aged ≥70 [42]. It has also been shown that Zostavax efficacy wanes relatively quickly (from 67.5% at <1 year post vaccination to 31.8% at 7–8 years post vaccination [43]). In contrast, the protection afforded by Shingrix is long-lasting (10+ years) [44]. However, Shingrix can cause severe local reactogenicity in up to 20% of recipients [45,46,47], and global rollout has been hampered by the scarcity of the adjuvant component QS-21 [48]. The high cost of Shingrix at approximately $200 per dose [49] may also affect global uptake of the vaccine.

Taken together, these observations suggest that there is still a need for alternative vaccines against RSV and HZ with improved tolerability, availability and cost, while also providing a high level of efficacy. As the target populations for these vaccines partially overlap, we endeavoured to develop a single vaccine against both RSV and HZ for use in older adults that would address these requirements.

Nonreplicating chimpanzee adenovirus (ChAd) vaccine vectors, such as ChAdOx1, have previously been shown to induce potent cellular and humoral immune responses, including CD8^+^ T cell responses not induced by other vaccine platforms [50]. The ChAdOx1 nCoV-19 vaccine (Vaxzevria) deployed during the COVID-19 pandemic offered excellent protection against disease alongside a good safety profile and was compatible with large scale global manufacture [51,52]. ChAdOx2 is a related adenoviral vector (based on Thichimpanzee adenovirus 68 (ChAd68), also known as SAdV-25 and Pan 9) [53]**,** whose safety and immunogenicity have been demonstrated in two Phase 1 trials [54,55]. In addition, process development and formulation work with the ChAdOx2 vector suggest feasibility of GMP manufacturing with excellent yields [56] and amenability to thermostabilisation [57]. Taking into consideration these favourable characteristics, ChAdOx2 was chosen as the basis for the development of our bivalent HZ and RSV vaccine candidate.

## 2. Materials and Methods

The RSV-A prefusion F (preF) protein sequence in the vaccine constructs was based on sequences identified in Joyce et al. [58]. Briefly, the p27 peptide (amino acids 104–142) of the RSV protein F was removed and replaced with a glycine linker to fuse the F1 and F2 fragments. In addition, several amino acid substitutions (S155C, S290C, S190F, V207L) were made to stabilize the antigenic site Ø and allow for the formation of stable trimers [59]. Additional changes (S46G, E92D, A149C, S215P, L373R, Y458C, K465Q) were made to increase RSV-protective responses (based on the Design Cycle 4 supplementary materials in Joyce et al. [58]). The resulting RSV preF DNA sequence was optimised for human codon usage.

The source of the VZV gE sequence was the VZV strain Oka gE protein (UniProtKB/Swiss-Prot Q9J3M8.1) [60]. The VZVgE DNA sequence was also optimised for human codon usage.

The amino acid sequence of the furin-2A site [61] was as follows: RRKRGSGEGRGSLLTCGDVEENPGP.

Two bivalent antigen cassettes were generated using conventional cloning techniques: RSVpreF-furin2A-VZVgE and VZVgE-furin2A-RSVpreF.

### 2.1. Generation of Vectors

The two bivalent antigen cassettes were flanked by the cytomegalovirus (CMV) immediate–early promoter and the bovine growth hormone polyadenylation (BGHpolyA) sequence and cloned into the ChAdOx2 E1 locus as described previously [53]. The resulting ChAdOx2-VZV-RSV and ChAdOx2-RSV-VZV vectors were rescued and propagated in a HEK293 tet repressor cell line, purified using the caesium chloride method and titrated using methods previously described [53]. All vector batches produced comparable titres, and particle to infectivity (P:I) ratios were between 60 and 200 for all batches, in line with expected values for ChAdOx vectors.

Monovalent control vectors were also generated using the same methods: The ChAdOx2-RSV vector encodes the RSV preF antigen only, while the ChAdOx1-VZVgE vector encodes the VZV gE antigen only [60]. ChAdOx1-VZVgE was chosen as the relevant monovalent control for VZV gE (rather than ChAdOx2), as this vector had already been characterised in previous preclinical studies [62].

### 2.2. Western Blot Analysis

**Figure 1:**
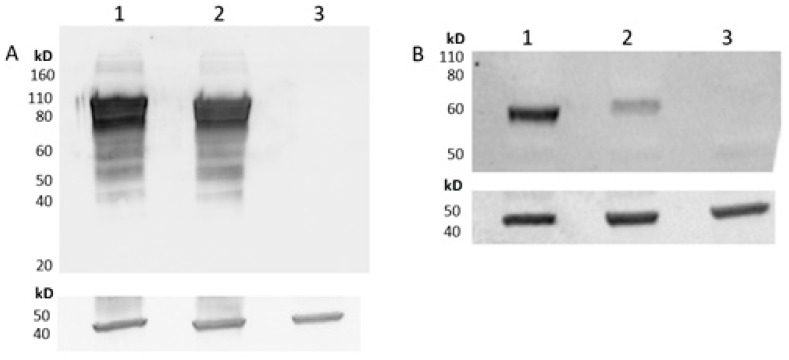
Expression of VZVgE (**A**) and RSVpreF (**B**) antigens in infected cell lysates as assessed via Western blot (upper panels). Lane 1: ChAdOx2-VZV-RSV, Lane 2: ChAdOx2-RSV-VZV, Lane 3: ChAdOx2-SEAP (negative control). Actin was used as a loading control (lower panels).

To confirm expression of VZVgE and RSVpreF antigens from the viral vectors, HEK293 cells were infected at an MOI of 1 or 5 IU and harvested after 48 h. A ChAdOx2 vector expressing secreted embryonic alkaline phosphatase (ChAdOx2-SEAP) was included as a negative control. Cell pellets were lysed with M-PER mammalian protein extraction reagent (ThermoFisher, Altrincham, UK) before being mixed with NuPAGE**^®^** LDS Sample Buffer and reducing agent (ThermoFisher), heat-denatured and run on a AnykD™ Criterion™ TGX™ Precast Midi Protein Gel (Biorad, Watford, UK). Proteins were transferred to nitrocellulose membranes using the iBlot system (Invitrogen, Paisley UK). Blots were probed with primary antibodies (anti-VZV gE (abcam (Cambridge, UK), ab272686), anti-RSV (abcam, ab12253) and anti-beta-actin (abcam, ab8224)) and relevant HRP-conjugated secondary antibodies. Pierce™ ECL Western Blotting Substrate (ThermoFisher) was used for chemiluminescence development. Blots were imaged using the BioRad Chemidoc system (Figure 1).

### 2.3. Mouse Study

Three groups of eight mice (female CD-1 mice, 8 weeks of age) and one group of three mice (control group) were vaccinated intramuscularly on day 0 and day 28. The dose for bivalent vectors (ChAdOx2-RSV-VZV and ChAdOx2-VZV-RSV) was 1 × 10^8^ IU per mouse and the monovalent mix contained 5 × 10^7^ IU each of ChAdOx1-VZVgE and ChAdOx2-RSV (for a total dose of 1 × 10^8^ IU per mouse). On day 42 (2 weeks post-boost) the mice were sacrificed, spleens were harvested and serum samples taken.

Each mouse group in this study was composed of 8 animals; however, viable splenocytes were only obtained from 6 mice each in groups 1 and 2 and 5 mice in group 3, due to technical challenges. Serum was obtained from all animals (*n* = 8 per group).

The mouse study was performed in accordance with the UK Animals (Scientific Procedures) Act 1986 at Charles River Discovery Research Services, Portishead, UK.

### 2.4. Cotton Rat Challenge Study

Three groups of 8 female cotton rats (6–8 weeks of age) were immunised intramuscularly (quadricep) with either PBS (group 1), ChAdOx2-RSV (group 2) or ChAdOx2-VZV-RSV (group 3) on days 0 and 28. The vector dose was 2 × 10^8^ IU per animal per immunisation, and blood was sampled on days 0, 28 and 49. All animals were challenged intranasally with 0.1 mL RSV/A/Long (Lot# 041513) at 1 × 10^5^ pfu per animal on day 49. On day 54, all animals were euthanised. Nasal and lung tissues were harvested in 3 mL of Hank’s Balanced Salt Solution (HBSS) with 10% sucrose-phosphate-glutamate and homogenised for viral titrations. Lung tissue was also placed in 10% neutral buffered formalin for histopathology (details below).

All cotton rat challenge work was conducted at Sigmovir (Rockville, MD, USA), which is accredited by the AAALAC (Association for Assessment and Accreditation of Laboratory Animal Care).

### 2.5. Immune Assays

#### 2.5.1. Enzyme-Linked Immuno-Spot Assay (ELISpot)

The cellular immune responses were assessed via interferon-γ (IFN-γ) ELISpot assay on fresh splenocytes as described previously [63]. Briefly, mouse spleens were passed through 70 mM cell strainers to produce splenocyte suspensions in RPMI medium, followed by treatment with ammonium chloride potassium (ACK) lysis buffer for red blood cell lysis. Splenocytes were then resuspended in mouse complete media (MCM, RPMI + GlutaMAX-1 + 10% FBS +10 µg/mL Gentamicin) and counted. Splenocytes (2 × 10^5^ per well in 100 µL of medium) were stimulated with peptide stimulants at a final concentration of 1 µg/peptide/mL in MCM in 96-well plates (Millipore, Gillingham, UK) which had previously been coated with Mouse IFN-γ ELISPOT Capture Antibody (BD™ ELISPOT Mouse IFN-γ ELISPOT Pair (Wokingham, UK). Cells were also treated with either 0.4% DMSO (negative control) or ConA (positive control). Plates were incubated at 37 °C in a 5% CO_2_ incubator overnight. Spot-forming cells (SFC) were detected with Mouse IFN-γ ELISPOT Detection Antibody (BD) followed by BD™ ELISPOT HRP Streptavidin for ELISPOT and BD™ ELISPOT AEC Substrate Set. Spots were counted using the AID ELISpot reader and software (Elispot 7.0). Negative control responses (from splenocytes cultured without peptide stimulation but with 0.4% DMSO) were subtracted from responses in peptide stimulated wells.

Peptides used for stimulation in the ELISpot assay were PepSet 15 mers (50–70% purity) offset by 4 amino acids (Mimotopes, Liverpool, UK), covering the RSV preF and VZVgE antigen sequences. Two peptide pools were generated for RSV preF, and three pools for VZVgE. RSV preF pool 1 consisted of peptides 1–67, covering the *N*-terminal half of the antigen, and RSV preF pool 2 consisted of peptides 68–135, covering the C-terminal half of the antigen. VZV gE pool 1 consisted of peptides 1–5 and 134–154, covering the signal peptide, transmembrane domain and cytoplasmic tail, VZV gE pool 2 consisted of peptides 6–69, covering the *N*-terminal half of the extracellular domain, and VZV gE pool 3 consisted of peptides 70–133, covering the C-terminal half of the extracellular domain.

#### 2.5.2. Enzyme-Linked Immunosorbent Assays (ELISA)

For the mouse study, humoral immune responses were assessed via ELISA using plates coated with either VZV gE protein (abcam ab43050) or human RSV Fusion glycoprotein (Sinobiological, Beijing, China, 11049-V08B) at 2 μg/mL. The secondary antibody in both cases was goat anti-mouse HRP conjugate (abcam ab5870). Serum samples were diluted (1:100, 1:300, 1:900, 1:2700: 1:8100 and 1:24,300) in duplicate and optical density at 450 nm (OD450) was measured after addition of TMB substrate (3,3′,5,5′-Tetramethylbenzidine (TMB) Liquid Substrate System for ELISA peroxidase substrate, Sigma, Gillingham, UK). The endpoint titres were calculated as the last dilution that gave a positive result on the ELISA. A positive result was defined as an OD450 value of at least 3 standard deviations above background. Samples whose OD450 never reached 3 standard deviations above background for any dilution step were classed as non-responders and assigned an arbitrary endpoint titre of 1 × 10^−1^ in order to include them in the graphs.

For the cotton rat challenge study, ELISA was performed as follows: Purified F protein extracted from RSV-infected Hep-2 cells was diluted and coated onto 96-well ELISA plates overnight. Plates were blocked (10% BSA Diluent/Blocking solution, KPL, 5140-0006) for one hour at room temperature and subsequently washed. Diluted sera (1:500 in duplicates) along with positive and negative controls were added to the wells and incubated at room temperature for one hour. After washing, plates were incubated with rabbit anti-rat IgG (1:500) (Sigmovir in-house reagent) for one hour at room temperature, followed by washing and incubation with goat anti-rabbit IgG-HRP (1:6000) (Merck, Gillingham, UK, AQ132P) for one hour at room temperature. Optical density at 450 nm (OD450) was measured after addition of TMB substrate.

### 2.6. RSV Plaque-Reduction Neutralisation Test

The neutralising antibody response against RSV was assessed using a plaque-reduction neutralisation test (PRNT). The assay was conducted by Sigmovir Biosystems Inc. (Rockville, MD, USA), using live RSV strain A2. Briefly, the serum to be assayed was heat-inactivated before undergoing serial dilution in Eagles Minimum Essential Medium (EMEM) (final dilution 1:20,480). The diluted samples were incubated with 25–50 PFU of live RSV strain A2 for one hour at room temperature before being added to confluent monolayers of Hep-2 cells in 24-well plates. The plates were incubated at 37 °C and 5% CO_2_ for one hour before being overlayed with 0.75% Methylcellulose medium. After four days at 37 °C and 5% CO_2_, cells were stained with 0.1% crystal violet stain to visualise and enumerate plaques. The reciprocal neutralising antibody titres were calculated at the 60% reduction end-point of the virus controls (statistics program used was plqrd.manual.entry).

### 2.7. Histopathology

Lungs were bisected, with left sections being assayed for RSV viral titre via PRNT and right sections being inflated with neutral buffered formalin for histopathology. Formalin fixed lungs were embedded in paraffin, sectioned and stained with hematoxylin and eosin (H&E). Pulmonary inflammation was evaluated using four parameters: peribronchiolitis (inflammatory cell infiltration around the bronchioles), perivasculitis (inflammatory cell infiltration around the small blood vessels), interstitial pneumonia (inflammatory cell infiltration and thickening of alveolar walls) and alveolitis (cells within the alveolar spaces). The tissues were blindly scored on a scale of 0 to 4, and results were subsequently converted to a 0–100% histopathology score. Scoring was as follows: 0: no lesions, 1: 5% severe lesions or up to 25% mild lesions, 2: 25% severe lesions or up to 75% mild lesions, 3: 75% severe lesions or up to 100% mild lesions and 4: 100% severe lesions.

### 2.8. RSV-A Lung and Nasal Viral Titrations

Lung and nose homogenates were clarified via centrifugation before being diluted in Eagles Minimum Essential Medium (EMEM) and used to infect confluent Hep-2 monolayers in duplicate in 24-well plates. The cells were incubated at 37 °C in a 5% CO_2_ incubator for one hour before being overlayed with 0.75% methylcellulose containing medium. After four days of incubation, the overlay was removed, and cells were fixed with 0.1% crystal violet stain for one hour before being rinsed and air dried. Viral plaques were counted, and the resulting viral titre is expressed as plaque-forming units per gram of tissue. Viral titres were calculated as geometric mean plus standard error for all animals in a group at a given time.

### 2.9. Statistical Analyses

**Figure 2:**
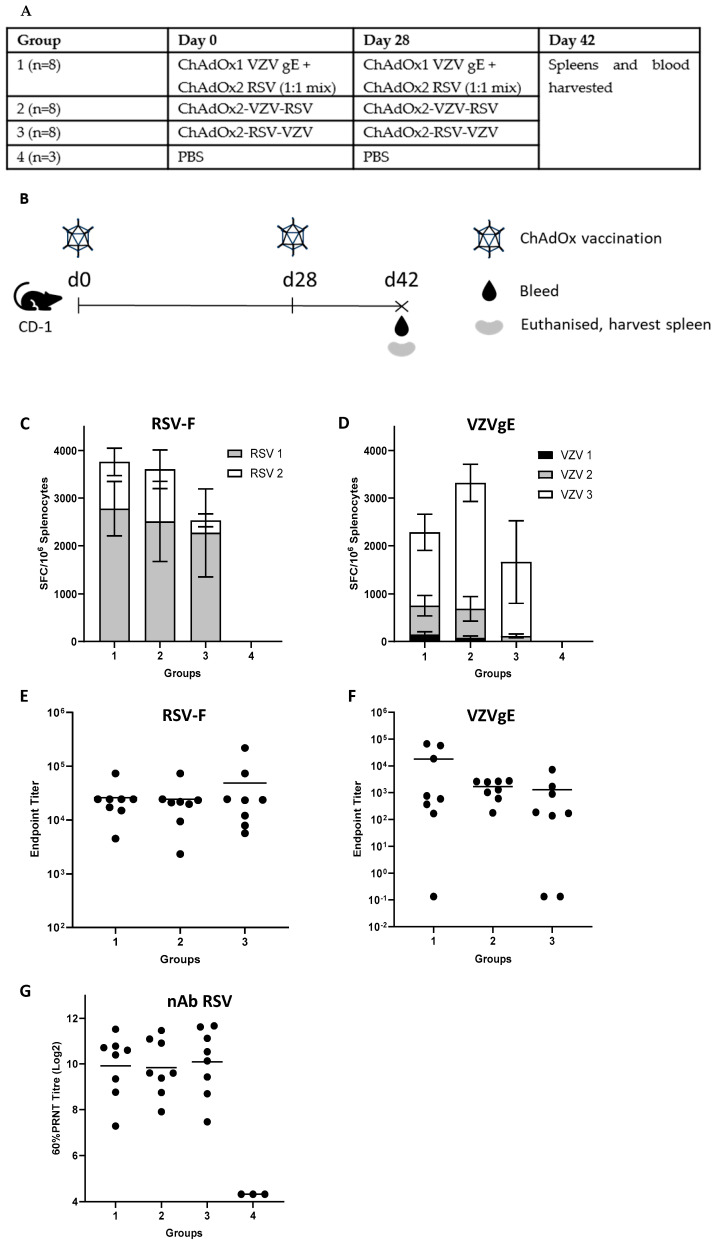
Mouse study to assess cellular and humoral immune responses after vaccination. (**A**,**B**) Experimental design: treatment groups and regimens. (**C**) T cell responses (as assessed via IFN-g ELISpot, mean with SEM per group) against RSVpreF peptide pools (RSV 1 and RSV 2). (**D**) T cell responses (as assessed via IFN-g ELISpot, mean with SEM per group) against VZVgE peptide pools (VZV 1, VZV 2, VZV 3). (**E**) Humoral immune responses (endpoint titres) as assessed via RSV-F ELISA. (**F**) Humoral immune responses (endpoint titres) as assessed via VZV gE ELISA. (**G**) Neutralising antibody (nAb) responses as assessed via RSV 60% plaque-reduction neutralising test (PRNT_60_) against live RSV strain A2. Horizontal lines represent mean values per group (**E**–**G**). No statistically significant differences were found between groups 1–3 in any datasets shown (**C**–**G**).

All statistical analyses were performed on GraphPad Prism 9.0. Means were used as representative values for each mouse group. Statistical comparisons were performed using one-way ANOVA on the following datasets: Figure 2C–G, groups 1–3. No statistically significant differences were found.

## 3. Results

### 3.1. Generation of Recombinant Viral Vectors and Confirmation of Antigen Expression

With the aim of creating a vaccine candidate that targets both RSV and HZ, we generated ChAdOx2 viral vectors expressing both the RSV F and VZV gE proteins. The RSV F protein is the main surface antigen involved in host–pathogen interactions and is well conserved among RSV subtypes. We chose to encode the RSV F protein in its prefusion conformation (preF), as this had previously been shown to induce higher neutralising antibodies than the unmodified F protein [64] and is also the antigenic target contained in Arexvy, the protein-in-adjuvant vaccine approved by FDA. The VZV antigen we selected was the full-length gE protein, the target of protective immunity in the highly efficacious Shingrix vaccine. Antigens were encoded in a single-expression cassette and linked via a furin-2A sequence [61] designed to result in translation of two separate, conformationally correct antigens. Two ChAdOx2 vectors were generated: one encoding RSV preF upstream of VZV gE (ChAdOx2-RSV-VZV) and vice versa (ChAdOx2-VZV-RSV), to enable selection of the best-performing vector.

Antigen expression from the ChAdOx2 vectors in a human cell line was confirmed via Western blotting. Robust expression of VZVgE and RSV preF from both vectors was apparent; RSV preF protein levels appeared slightly lower when expressed from the ChAdOx2-RSV-VZV vector (Figure 1B). The multiple bands observed in the VZVgE blot (Figure 1A) likely reflect different glycosylation levels of the gE protein [65]. Expression levels of VZV gE were equivalent from both vectors (Figure 1A).

### 3.2. Immunogenicity in Mice

Next, we assessed the immunogenicity of the bivalent vaccine candidates in mice and compared the results against the immune response generated by a mixture of monovalent vectors. A homologous prime/boost experiment in CD-1 outbred mice was performed as outlined in Figure 2A,B. Group 1 was vaccinated with a 1:1 mixture of the monovalent vectors ChAdOx1-VZVgE and ChAdOx2-RSV. Group 2 was vaccinated with ChAdOx2-VZV-RSV, group 3 with ChAdOx2-RSV-VZV and group 4 with PBS (days 0 and 28). At day 42 (two weeks post-boost, at peak T cell and antibody response [66]), mice were euthanised and spleens and serum taken for ELISpot and ELISA, respectively, to assess the cell-mediated and humoral immune response to the antigens. ELISpot results demonstrated that ChAdOx2-VZV-RSV outperformed ChAdOx2-RSV-VZV in terms of IFN-γ T cell response (Figure 2C,D): the summed average T cell responses against RSV preF (Figure 2C) as well as the summed average T cell responses against VZVgE (Figure 2D) were higher in group 2 compared to group 3, although these differences were not statistically significant. The ChAdOx2-VZV-RSV T cell response also compared favourably to the 1:1 mix of the two vectors. The average antibody responses to VZVgE were similar for the two bivalent vectors, although ChAdOx2-RSV-VZV did not induce antibodies in 2 out of 8 mice (Figure 2F). The monovalent mix induced slightly higher average VZVgE endpoint titres, but this difference was not statistically significant (Figure 2F). The antibody responses to RSV (Figure 2E) were similar in all three groups.

Serum from the mouse experiment was also used in an RSV plaque-reduction neutralisation test (PRNT) using live RSV-A2 virus. All vector regimens produced similar levels of neutralising antibodies against RSV (Figure 2G), reproducing the observation of comparable total antibody levels seen via ELISA (Figure 2E).

Based on the combined mouse immunogenicity data, the ChAdOx2-VZV-RSV vector was selected as the preferred vector candidate for further preclinical characterisation, as it trended toward outperforming the other bivalent vector with regard to T cell induction against both antigens (although neither difference was statistically significant).

### 3.3. Cotton Rat RSV Challenge

**Figure 3:**
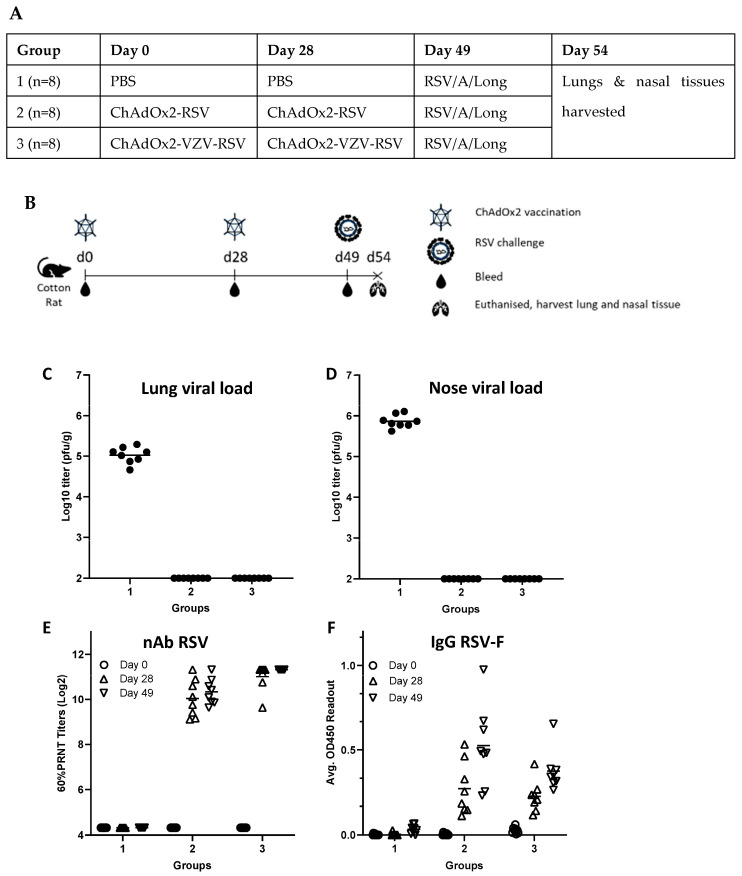
Cotton rat challenge study to assess vaccine efficacy against RSV infection. (**A**,**B**): experimental design. Protection after RSV challenge as assessed via lung viral load (**C**) and nose viral load (**D**) measured in pfu/g of tissue (limit of detection 2.0 Log10 pfu/g). (**E**) Neutralising antibody (nAb) responses as assessed via RSV 60% plaque-reduction neutralising test (PRNT_60_) against live RSV strain A2 (lower limit of detection 4.32 Log2 and upper limit of detection 11.32 Log2). (**F**) Humoral immune responses as assessed via RSV-F ELISA (OD450). Horizontal lines represent mean values per group (**C**–**F**).

In order to evaluate the efficacy of our preferred candidate, we assessed the ability of the ChAdOx2-VZV-RSV vector to protect against RSV infection in a cotton rat challenge model, a commonly used animal model for preclinical studies of RSV infection and vaccine-induced protection [67,68]. Monovalent ChAdOx2-RSV was included as a control to compare the efficacy of monovalent and bivalent vectors. As indicated in Figure 3A, cotton rats received either PBS (group 1), ChAdOx2-RSV (group 2) or ChAdOx2-VZV-RSV (group 3) on days 0 and 28. The animals were then challenged on day 49 with RSV/A/Long before being sacrificed at day 54, with blood, nasal tissue and lungs taken for further analysis (Figure 3B).

As a primary efficacy readout, the RSV viral load in the lungs and nasal tissue of the animals was determined at 5 days post-infection. No live RSV was detected in the lungs or noses of cotton rats vaccinated with either ChAdOx2-RSV or ChAdOx2-VZV-RSV, whereas high levels of infectious virus were found in unvaccinated animals (Figure 3C,D respectively).

A PRNT assay was performed on serum taken on days 0, 28 and 49 to assess RSV-neutralising antibody activity before and after immunisation. Baseline serum on day 0 showed no neutralisation in any of the animals, as expected. Cotton rats vaccinated with ChAdOx2-RSV or ChAdOx2-VZV-RSV displayed high levels of neutralising antibodies against RSV on days 28 and 49, with a trend for higher levels on day 49 compared to day 28, while unvaccinated animals showed no detectable levels of anti-RSV neutralising antibodies (Figure 3E).

In addition to PRNT, total IgG antibody responses to the vectors were measured via RSV ELISA. Anti-RSV antibodies were detected at day 28 with an increase above levels detected on day 49 (Figure 3F). There was no anti-RSV antibody response in those cotton rats that received PBS (group 1), as expected.

**Figure 4:**
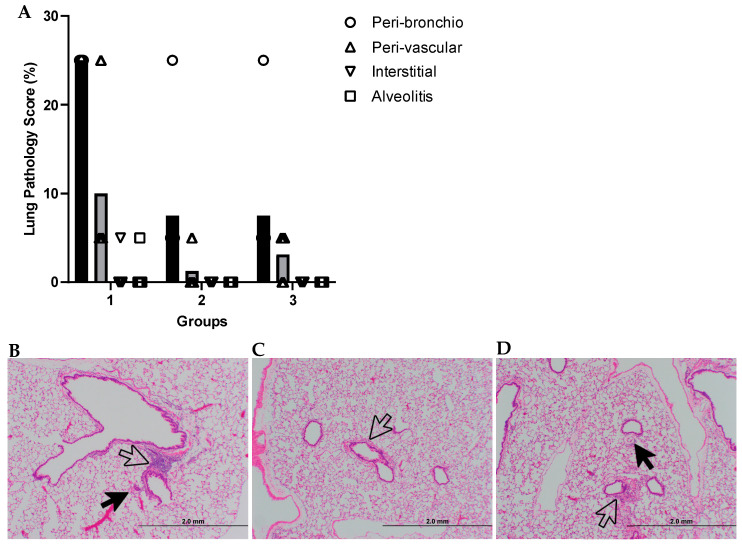
(**A**) Histopathology scoring of cotton rat lungs after RSV challenge (see Section 2 for details; score represents percentage of lung tissue affected by each type of inflammation assayed). Groups and treatments as described in Figure 3A,B. Group 1: PBS. Group 2: ChAdOx2-RSV. Group 3: ChAdOx2-VZV-RSV. Grey columns represent mean scores per group. (**B**–**D**) Representative lung histopathology images from one animal per group. (**B**) Group 1 animal with a peri-bronchio score of 25 and a peri-vascular score of 5 (all other scores 0). (**C**) Group 2 animal with a peri-bronchio score of 5 (all other scores 0). (**D**) Group 3 animal with a peri-bronchio score of 5 and a peri-vascular score of 5 (all other scores 0). Filled black arrows indicate perivasculitis; open arrows indicate peribronchiolitis.

A previous RSV vaccine candidate (formalin-inactivated) had been shown in the 1960s to cause enhanced disease (weight loss, pulmonary inflammation, mucus hypersecretion and airway obstruction) in children subsequently infected with RSV [69]. We therefore tested whether there were any signs of disease enhancement caused by our vectors. Lung tissue of all animals was fixed and stained with hematoxylin and eosin (H&E) to determine pulmonary histopathology at 5 days post RSV challenge. Four types of inflammation were evaluated and scored for severity: peribronchiolitis, perivasculitis, interstitial pneumonia and alveolitis. All control animals (group 1) showed signs of peribronchiolitis with low levels of perivasculitis, interstitial pneumonia and alveolitis, as expected after RSV infection of the lower respiratory tract. Animals vaccinated with ChAdOx2-RSV (group 2) or ChAdOx2-VZV-RSV (group 3) showed some peribronchiolitis (one outlier in each group) and perivasculitis, but no interstitial pneumonia or alveolitis (Figure 4). These results suggest that our vaccine candidates do not cause disease enhancement upon RSV challenge.

## 4. Discussion

In this report we show that a bivalent vaccine candidate, ChAdOx2-VZV-RSV, elicited robust cellular and humoral responses against VZV gE and RSV preF protein in outbred mice and was protective in a cotton rat RSV challenge model. A two-shot (rather than single-shot) immunisation regimen was chosen based on clinical study results with a ChAdOx1-based COVID-19 vaccine (Vaxzevria), which showed that antigen-specific antibody and B cell responses were significantly boosted after the second administration of the viral vector [70].

After prime/boost immunisation with our bivalent vector candidate, the antigen-specific immune responses seen in mice were similar to those observed for a mixture of monovalent vectors, indicating that encoding two antigens in the same vector does not result in diminished immunogenicity. As larger antigenic cargo can cause genetic instability in adenoviral vectors, we also assessed the bivalent vectors for any apparent signs of genetic instability. No genetic changes in the vector genomes or reduced vector yield were observed during the generation of small-scale vector batches, suggesting that both bivalent vectors are genetically stable, although the gold standard assay, a formal genetic stability study over 10 viral passages, remains to be performed.

The authors acknowledge that the animal studies presented here have several limitations. While the studies included control groups that received PBS, there were no control groups that received empty vector (or vector encoding an irrelevant protein). PBS groups allow for controlling for any effects associated with the administration procedure itself, while using an empty vector would additionally control for any effects related to the viral vector itself that are not antigen-specific. In the cotton rat challenge study, for example, an empty vector control would allow for assessing whether the protective effect is solely due to the antigen-specific immune response, or whether a vector-induced innate response (elicited by the vector alone) might also have a role in protection. Innate responses elicited by adenoviral vectors have been shown to be short-lived [71] and would not be present at the time of RSV challenge (i.e., 3 weeks after the last immunisation); they are therefore not expected to have an impact on post-challenge responses in our study. Another limitation of our studies is the absence of demonstration of reproducibility across studies and across batches of test material, as each study was only performed once, with the same test material. However, positive and negative internal controls were used in each study, and reproducibility was shown for certain data sets, namely the induction of similarly high levels of neutralising antibodies against RSV with our preferred vaccine candidate in both mouse and rat species (Figure 2G and Figure 3E).

There is no generally accepted correlate of protection for HZ. However, the highly efficacious Shingrix vaccine induces strong anti-gE-specific humoral and polyfunctional CD4^+^ responses (with no detectable CD8^+^ T cells), and the proposed correlate of protection for this vaccine is frequency of antigen-specific polyfunctional CD4^+^ T cells that express two or more cytokines. While antibody levels did not correlate with efficacy for Shingrix, they are likely still important. Our preclinical immunogenicity studies showed robust antibody and T cell induction against the VZV gE antigen. While we did not perform further immunophenotyping of this response in this report, a previous study with the monovalent vector ChAdOx1-VZVgE showed that it compared favourably with Shingrix [62]. This ChAdOx1-based HZ vaccine candidate has recently been approved to enter a Phase 1 trial in Canada to assess its safety and immunogenicity in older adults. VZV is highly species-specific (naturally only infecting humans and great apes) [72], and no appropriate small animal model exists for HZ [73]. An animal model using guinea pigs is only possible with the use of a guinea pig-adapted strain of VZV [72]. We were therefore unable to preclinically assess the efficacy of our vaccine candidate in preventing HZ.

A recent study evaluating the correlates of protection against RSV in a nonhuman primate model found that upper respiratory control of viral infection was associated with virus-specific IgA levels, neutralisation and complement activity, whereas lower respiratory control was associated with Fc-mediated effector mechanisms such as NK cell-mediated antibody-dependent cellular cytotoxicity (ADCC) [74]. The immune correlates that have been assessed for protection from Arexvy, which is approved for the prevention of lower respiratory tract disease caused by RSV in individuals 60 years of age and older, are total antigen-specific IgG levels as well as neutralising antibody levels [8]. Our preclinical studies showed induction of robust total antibody as well as neutralising antibody levels against RSV and protection in a cotton rat RSV challenge after two administrations of our viral vector-based vaccine. In addition, no disease enhancement was apparent after immunisation and RSV challenge in cotton rats, suggesting that our vector-based candidate is safer than previously assessed formalin inactivated vaccines [69].

The induction of an anti-vector immune response is a well-described phenomenon associated with viral vectored vaccines. Anti-vector neutralising antibodies (nAb) are observed after a single adenovirus vector immunisation, and these nAb levels are maintained or boosted via repeat administration of the same adenoviral vector. High anti-vector nAb levels have been proposed to negatively impact antigen-specific immune responses after a subsequent immunisation with the same vector, although recent studies report no or only a slight negative correlation between pre-boost nAb levels and post-boost antigen-specific antibody responses [75,76,77]. In light of the global roll-out of the ChAdOx1-based COVID-19 vaccine (ChAdOx1-nCoV), it will be important to assess whether there is an impact of anti-ChAdOx1 vector immunity on alternative chimpanzee-based adenoviral vaccine candidates such as the ChAdOx2 vector used in this study. A previous study reported no or very low cross-reactivity against ChAdOx1 in serum from ChAdOx2-immunised trial participants [54], suggesting that these vectors are structurally different enough to circumvent cross-neutralisation. In addition, it has been shown that anti-vector immunity wanes with time [76] and likely drops to levels that are no longer clinically relevant within several months after immunisation. This is supported by reports that a longer boosting interval between ChAdOx1-nCoV administrations (3 months compared to 4 weeks) improved boosting response and vaccine efficacy [78], as well as observations that prior immunisation with unrelated ChAdOx1-based vaccine candidates did not affect the immune responses to the ChAdOx1-nCoV vaccine [79].

In developing our rationale for advancing this vaccine candidate, we consider several factors to be highly supportive, including availability of materials needed for manufacture, cost of goods and reactogenicity. Addressing the former, it is important to note that one of the components of the AS01 adjuvant contained in both Shingrix and Arexvy is QS-21, a saponin that is isolated from *Quillaja saponaria* tree bark and is a potent activator of a Th1-type immune response. However, despite ongoing efforts to understand the chemically complex biosynthesis pathway and to develop synthetic QS-21 and alternative synthetic saponins [80], the only current commercial source of QS-21 is the soapbark tree native to central Chile. This brings with it a number of challenges such as scarcity of natural habitats and variable levels of QS-21 per tree, leading to potential limits on total QS-21 amounts that can be commercially produced.

Another consideration is cost of goods, with Shingrix currently priced at US $200 per dose (and price discussions for Arexvy still ongoing but likely to be in a similar range). In contrast, large-scale manufacturing of adenoviral vectors is highly cost-effective, with Vaxzevria, for example, being sold at US $2–6 per dose. We therefore envision a low cost of goods for our vaccine candidate, which would benefit low- and middle-income countries (LMIC) in particular.

Lastly, we considered potential benefits of our candidate in terms of reactogenicity. Here, once more, we based our predictions for our vaccine candidate on Vaxzevria, as our candidate makes use of a similar platform technology, and reactogenicity is thought to be driven primarily by the adjuvanting effect of the vaccine modality. (Of note, Arexvy contains the same (AS01) adjuvant as Shingrix.) Based on European public assessment reports (EPAR) information, the type and frequency of mild and moderate adverse reactions are comparable between Vaxzevria and Shingrix, with both vaccines being associated with systemic adverse effects such as fatigue, headache and myalgia [81,82]. However, compared to Vaxzevria, Shingrix causes a significantly higher percentage of severe local adverse reactions: pain, redness and swelling of the injection site were experienced by about 10% of adults who received Shingrix [83], while only 1.7% of subjects experienced severe local reactions after Vaxzevria [84]. The reactogenicity seen with Shingrix may have an impact on second-dose completion, with non-completion rates of 20% having been reported in one study [85]. We believe that our RSV VZV vaccine candidate would likely have a similar reactogenicity profile as Vaxzevria, with fewer severe local effects compared to Shingrix.

In summary, the favourable (albeit limited) preclinical immunogenicity and efficacy profile of our candidate together with the limitations of the current licensed RSV and shingles vaccines support our rationale for the further clinical development of our viral vector-based RSV and shingles vaccine candidate, which does not rely on adjuvants, is likely to cause less severe local reactogenicity and can be manufactured at a low cost per dose.

## Data Availability

Data is available from the corresponding author on request.
